# Calcium Sulphide in the Treatment of Diabetes Mellitus

**Published:** 1884-04-20

**Authors:** C. M. Caldwell

**Affiliations:** Visiting Physician to St. Joseph’s Hospital


### CALCIUM SULPHIDE IN THE TREATMENT OF DIABETES MELLITUS.

By C. M. CALDWELL, M.D.,

Visiting Physician to St. Joseph's Hospital.

Within the past ten years so-called calcium sulphide has been recommended by several observers as a valuable drug in the treatment of diabetes mellitus. Dr. N. C. Husted, who was himself afflicted with this disease, made a complete and lasting recovery by using the drug in connection with appropriate hygienic treatment. The doctor has since employed it with satisfaction in other cases of diabetes.

Dr. Austin Flint, Sr., has prescribed the remedy, along with suitable diet, general management, etc., in several cases which progressed favorably.

Dr. C. H. Lellman gave sulphide of calcium with remarkably good effect in a single case occurring in his service at St. Francis's Hospital in 1880.

I have used the drug in only three cases. In one it produced no effect whatever. In the others, improvement began and recovery took place during the administration of the remedy.

The following are brief histories of the cases which ended in recovery:

CASE I.—Mr. H. B., aged sixty-five, an artist. First seen on February 20, 1883. For five or six years he had suffered from severe attacks of asthma, but, until within two years, his general health was good, although financial difficulties caused much mental depression and worry. During the past eighteen months he had noticed a slow, but steady increase in the amount and frequency of micturition at night. Dryness of the mouth and throat, with marked thirst, had gradually become very troublesome, and progressive loss of strength and ambition had been observed by his friends. By far the most prominent symptom, however, was his enormous appetite—as he expressed it, "he ate like a starving wolf, yet remained hungry night and day." Sight had not notably failed. There had been no cutaneous complication, and the bowels had remained regular.

Examination showed considerable pulmonary emphysema, slight bronchitis and marked anæmia. Skin dry. Extremities cold. Saliva faintly acid. Urine pale, acid, sp. gr. 1·042, and a strong sugar reaction with Squibb's Fehling's solution. A moderately strict "diabetic diet," daily out-of-door exercise, with iron and one-quarter of a grain of calcium sulphide five times daily, were prescribed.

February 22d.—Patient passed 185 oz. of urine to-day. Sp. gr. 1·038. Sugar abundant.

23d.—Passed 200 oz. Sp. gr. 1·040. Sugar abundant.

24th.—Passed 150 oz. Sp. gr. 1·040. Sugar abundant.

25th.—Passed 168 oz. Sp. gr. 1·038. Sugar abundant.

March 1st.—Patient's general condition has improved slightly during the past week. Appetite is less ravenous, and thirst not so intense. Passed 100 oz. Sp. gr. 1·036. Sugar abundant.

7th.—A steady improvement has been noticed in the more annoying symptoms. Night before last the patient had a severe attack of asthma, presumably due to an exacerbation of his chronic bronchitis, from which he is now suffering. Passed 69 oz. Sp. gr. 1·030. Sugar abundant. Ordered an expectorant mixture for bronchitis, and fluid extract of quebracho should asthma return.

14th.—Continuous improvement in the diabetic symptoms, but there were two attacks of asthma last week. Passed 63 oz. Sp. gr. 1·034. Sugar present.

22d.—Improvement continues. Amount of urine not ascertained. Sp. gr. 1·028. Sugar present.

April 2d.—Excessive thirst and hunger, with frequent calls to micturate, have ceased to annoy. An appreciable gain in flesh and spirits has taken place within two weeks. Passed 60 oz. Sp. gr. 1·020. No sugar.

4th.—Passed 53 oz. Sp. gr. 1·026. Trace of sugar.

15th.—For the past ten days the urine has contained no sugar, the sp. gr. varying between 1·020 and 1.030. The diabetic symptoms have subsided.

October 22d.—The patient was seen to-day for the first time in five months. During that period he has had no return of his former symptoms. A specimen of his urine gave an acid reaction, sp. gr. 1·016, and contained no sugar.

CASE II.—Mr. S. S., aged twenty-four, leather dresser. Came under observation May 22, 1883. The patient had been healthy and fairly robust until about one year ago, when he began to lose flesh and strength, to have continual thirst, irregular appetite, flatulent dyspepsia, persistent constipation, repeated headache and backache, and frequent micturition accompanied by urethral smarting. There had never been chills or sweating during this period. The above symptoms continued, although much modified for a time by treatment. During the past four months a dry morning cough, occasionally accompanied by "stitch-like" pains in the right side of chest, with slight dyspnœa on exercise, had been added to the other symptoms. The young man gave no family history of phthisis, but his business had necessitated his remaining in a dusty atmosphere for many hours each day.

Physical examination showed consolidation of the right apex anteriorly, a considerably enlarged spleen, dry skin, with patches of eczema on the lower extremities and genital organs. The mouth and throat were red and irritated, but not particularly dry. Saliva neutral in reaction. Urine clear, pale, acid, sp. gr. 1·038, strong sugar reaction with Fehling's solution. A diabetic diet, cod-liver oil, iron, and sulphide of calcium in one-half-grain doses three times daily, were ordered. Also, a laxative pill and regular exercise in the open air.

May 23d.—Passed 165 oz. of urine to-day. Sp. gr. 1·049. Sugar abundant.

29th.—During the last five days the daily amount of urine passed has varied between 170 and 150 oz., the sp. gr. ranging from 1·040 to 1·035.

June 8th.—Slight improvement has taken place in his general condition. The diabetic symptoms are less annoying and the digestion markedly improved. Urine not measured. Sp. gr. 1·035. Sugar abundant.

15th.—Passed 70 oz, Sp. gr. 1·030. Sugar present. Patient is improving steadily.

21st.—Passed 72 oz. Sp. gr. 1·030. No sugar.

30th.—Urine not measured. Sp. gr. 1·025. No sugar present. Diabetic symptoms have disappeared, but evidences of the lung trouble are present to the same extent as formerly.

July 25th.—The urine has been examined frequently since the last note, and has always been free from sugar, but, as the symptoms and physical signs of lung consolidation did not improve, the patient was advised to start for Switzerland at once. He did so a week ago.

October 22d.—Nothing further has been learned concerning this patient.

It will be observed that in the first case improvement commenced in two weeks, but the sugar did not entirely disappear until six weeks after treatment was begun.

In the second case benefit was unquestionable at the end of three weeks, and no sugar was detected after one month of treatment. The patient with whom calcium failed took the drug faithfully for one month.

Although calcium sulphide is certainly not a specific in diabetes, yet it seems worthy of a trial in persistent cases of this distressing disease.—*New York Med. Jour.*

